# Biomechanical Determinants of Performance and Injury Risk During Cutting: A Performance-Injury Conflict?

**DOI:** 10.1007/s40279-021-01448-3

**Published:** 2021-04-03

**Authors:** Thomas Dos’Santos, Christopher Thomas, Alistair McBurnie, Paul Comfort, Paul A. Jones

**Affiliations:** 1 Department of Sport and Exercise Sciences, Musculoskeletal Science and Sports Medicine Research Centre, Manchester Metropolitan University, All Saints Building, Manchester Campus John Dalton Building, Manchester Campus, Manchester, M15 6BH UK; 2 Human Performance Laboratory, Directorate of Sport, Exercise, and Physiotherapy, University of Salford, Salford, Greater Manchester UK; 3 Manchester United Football Club, Manchester, UK

## Abstract

**Background:**

Most cutting biomechanical studies investigate performance and knee joint load determinants independently. This is surprising because cutting is an important action linked to performance and non-contact anterior cruciate ligament (ACL) injuries. The aim of this study was to investigate the relationship between cutting biomechanics and cutting performance (completion time, ground contact time [GCT], exit velocity) and surrogates of non-contact ACL injury risk (knee abduction [KAM] and internal rotation [KIRM] moments) during 90° cutting.

**Design:**

Mixed, cross-sectional study following an associative design. 61 males from multidirectional sports performed six 90° pre-planned cutting trials, whereby lower-limb and trunk kinetics and kinematics were evaluated using three-dimensional (3D) motion and ground reaction force analysis over the penultimate (PFC) and final foot contact (FFC). Pearson’s and Spearman’s correlations were used to explore the relationships between biomechanical variables and cutting performance and injury risk variables. Stepwise regression analysis was also performed.

**Results:**

Faster cutting performance was associated (*p* ≤ 0.05) with greater centre of mass (COM) velocities at key instances of the cut (*r* or *ρ* = 0.533–0.752), greater peak and mean propulsive forces (*r* or *ρ* = 0.449–0.651), shorter FFC GCTs (*r* or *ρ* = 0.569–0.581), greater FFC and PFC braking forces (*r* = 0.430–0.551), smaller hip and knee flexion range of motion (*r* or *ρ* = 0.406–0.670), greater knee flexion moments (KFMs) (*r* = 0.482), and greater internal foot progression angles (*r* = − 0.411). Stepwise multiple regression analysis revealed that exit velocity, peak resultant propulsive force, PFC mean horizontal braking force, and initial foot progression angle together could explain 64% (*r* = 0.801, adjusted 61.6%, *p* = 0.048) of the variation in completion time. Greater peak KAMs were associated with greater COM velocities at key instances of the cut (*r* or *ρ* = − 0.491 to − 0.551), greater peak knee abduction angles (KAA) (*r* = − 0.468), and greater FFC braking forces (*r* = 0.434–0.497). Incidentally, faster completion times were associated with greater peak KAMs (*r* = − 0.412) and KIRMs (*r* = 0.539). Stepwise multiple regression analysis revealed that FFC mean vertical braking force and peak KAA together could explain 43% (*r* = 0.652, adjusted 40.6%, *p* < 0.001) of the variation peak KAM.

**Conclusion:**

Techniques and mechanics associated with faster cutting (i.e. faster COM velocities, greater FFC braking forces in short GCTs, greater KFMs, smaller hip and knee flexion, and greater internal foot progression angles) are in direct conflict with safer cutting mechanics (i.e. reduced knee joint loading, thus ACL injury risk), and support the “performance-injury conflict” concept during cutting. Practitioners should be conscious of this conflict when instructing cutting techniques to optimise performance while minimising knee joint loading, and should, therefore, ensure that their athletes have the physical capacity (i.e. neuromuscular control, co-contraction, and rapid force production) to tolerate and support the knee joint loading during cutting.

**Supplementary Information:**

The online version contains supplementary material available at 10.1007/s40279-021-01448-3.

## Key Highlights

**Table:** 

Techniques and mechanics associated with faster cutting performance are in direct conflict with safer cutting mechanics (i.e. reduced knee joint loading), and support the “performance-injury conflict” concept that is present during cutting.
Practitioners must be cautious when coaching and manipulating cutting technique and mechanics, and acknowledge the implications of technique modification on performance and potential injury risk.
Practitioners are encouraged to coach penultimate foot contact dominant braking strategies and minimising knee valgus and lateral trunk flexion to facilitate effective performance and potentially reduce knee joint loading.

## Introduction

An athlete’s ability to change direction is one of the most important physical qualities for successful performance in multidirectional sports [1–8], and is considered to provide the mechanical foundation for efficacious agility performance [3, 5, 9–11]. Change of direction (COD) manoeuvres are frequently performed in sports, such as soccer [4, 6], netball [1, 12], and rugby [13–15], with soccer players performing ~ 600 cuts of 0°–90° [6] during match play, while directional changes of 45° and 90° are frequently performed actions in netball [1]. Specifically, side-step cutting actions are the most commonly performed attacking agility action in netball [12], and are typically performed to create separation from an opponent to get into space and receive a pass. Moreover, side-steps are successful evasive manoeuvres in rugby and are linked to positive outcomes such as penetrating the defensive line [13, 14, 16]. As such, developing an athlete’s side-step mechanical cutting ability can be considered an important attribute to develop first, particularly from a motor skill learning perspective, before then incorporating unanticipated stimulus within practice drills to better prepare athletes for the chaotic demands of multidirectional sports [5, 9, 17, 18].

Changing direction, particularly side-step manoeuvres, has been identified as a key action associated with non-contact ACL injuries in numerous multidirectional sports (soccer, rugby, handball, netball, Australian rules football, American football, and badminton) [19–28], due to the potential to generate high multiplanar knee joint loading (flexion, rotation, and abduction moments) during the plant foot contact [29–33], thus increasing ACL strain [34–38]. ACL injuries are debilitating and potentially career threatening, with short- and long-term consequences (financial, health, and psychological) [39–43]. Specifically, an elevated and earlier risk of developing osteoarthritis is a primary concern associated with ACL injury [42, 44]. An estimated 2 million ACL injuries occur worldwide [45], most of which typically require surgery [46]; thus, extensive rehabilitation periods are required, resulting in prolonged absence from sport and the potential to lose sporting scholarships or contracts [47]. However, athletes who do successfully return to sport post ACL reconstruction may demonstrate reduced sports-related performance, reduced number of appearances, and shorter career longevity [48, 49]. Therefore, understanding the mechanics and techniques that can reduce the relative risk of injury during COD actions, while improving performance, are of great interest to researchers and practitioners working with multidirectional athletes.

Despite the importance of directional changes for sports performance and its association with ACL injury risk, it is somewhat surprising that the majority of studies into COD biomechanics investigate performance [50–60] and ACL injury risk surrogate determinants [30, 31, 33, 61–69] independently. From a performance perspective, greater braking and propulsive forces and impulses over short GCTs are related to faster COD speed performance [50, 51, 53–56, 58–60, 70]. Additionally, whole-body kinetics and kinematics such as greater ankle power, ankle plantar-flexor moments, hip power and extensor moments, rapid knee and hip extension, wide lateral foot plants, torso lean and rotation, and low COM are also associated with faster cutting performance [50, 53, 70]; highlighting the importance of the lower-limb triple extensor musculature and trunk lean towards the intended direction of travel. Conversely, from an injury risk perspective, COD techniques with a wide lateral foot plant [31, 33, 62, 70], greater hip abduction angles [52, 68], increased initial foot progression angles [61, 68], increased initial hip internal rotation angles [63, 64, 68, 70], greater peak and initial KAA [33, 61–64], greater lateral trunk flexion [31, 62, 67, 71, 72], smaller knee flexion angles [52, 73], and greater ground reaction forces (GRF) [30, 63, 68] are associated with greater peak KAMs and thus greater ACL strain [35, 74–77]. However, less is known regarding the mechanics and techniques necessary for optimal COD performance and how they relate and interact with injury risk [70, 78, 79].

There is preliminary evidence, although limited, which indicates the techniques and mechanics required for faster COD performance are in direct conflict with the techniques and mechanics required for safer COD (i.e. lower knee joint loads) [70, 78–81]. For instance, COD techniques such as increased IFPAs and pelvic and hip internal rotation angles are associated with greater KAMs [31, 61, 68], but may be optimal for COD performance due to effective realignment of the whole-body COM into the new intended direction [61, 82]. Extended knee postures (i.e. smaller knee flexion) increase anterior tibial shear and subsequently strains the ACL [74, 83–87], yet increasing knee flexion during side-stepping increases GCT and reduces exit velocity [80], thus negatively affecting performance. Greater KFMs [70] and posterior GRF [57, 58, 88] are associated with faster COD performance, but can also increase proximal anterior tibial shear [87, 89] and potential ACL loading [74, 83–85]. Lateral trunk flexion has been shown to increase knee joint loading [31, 32, 62]; however, this strategy may be adopted by athletes to deceive (feint) opponents [90–92]. Wide lateral foot plants [31–33, 62, 70] are also associated with greater KAMs, where larger moment arms and KAMs are created with a more medial whole-body position with respect to the foot and centre of pressure positioning more lateral to the COM of the body and tibia [61, 70]. However, a wide lateral foot plant is required for medial–lateral GRF and impulse generation to accelerate into the new direction [53, 62, 70, 93].

To the authors best knowledge, Havens and Sigward [70], Sankey et al. [79], and McBurnie et al. [81] are the only researchers to investigate the biomechanical determinants of cutting performance and surrogates of ACL injury risk, confirming that techniques required for faster performance are in direct conflict with reduced knee joint loading. For example, Havens and Sigward [70] revealed faster cutting performance was associated with greater lateral foot plant distances, medial–lateral impulse, and internal hip rotation angles, though it is worth noting that greater KAMs were also observed with wider lateral foot plants, which may increase ACL injury risk. Additionally, Sankey et al. [79] found increases in sagittal triple acceleration, frontal plane hip acceleration and transverse plane hip acceleration were related to sharper COD angles, while sagittal triple acceleration also related to greater medial COM acceleration; however, the aforementioned variables were also associated with greater KAMs. McBurnie et al. [81] observed greater peak KAMs and KIRMs were demonstrated by athletes who demonstrated faster cutting completion times, greater horizontal approach velocities, and greater peak hip flexion moments. Moreover, a recent review by Fox [78] has highlighted that reducing “high-risk” postures (such as wide foot plants, lateral trunk flexion, increasing knee flexion, internal hip and foot progression angle) are viable strategies to reduce such knee joint loads, but could be to the detriment of faster performance. As athletes are driven by performance, they may be unlikely to adopt movement strategies which decrease knee injury risk if they do not result in effective performance [70, 78]. Collectively, these studies suggest that there is a “performance–injury conflict” during COD, which is problematic for practitioners who aim to improve their athletes’ performance and reduce injury risk. As such, further insight is required to improve our understanding of mechanics required for faster and safer COD.

Although previous work has indeed provided further insight into the performance and injury risk determinants during cutting [70, 79], McBurnie et al. [81] is the only study to consider KIRMs while also examining PFC braking characteristics. This is important because ACL strain is amplified when a combination of high frontal and transverse knee moments are generated in comparison to uniplanar loading [34–38], and emerging research has demonstrated that greater braking forces displayed during the PFC (i.e. PFC dominant braking) is associated with faster COD performance [57, 58, 88, 94] and reduced KAMs in the FFC [30, 61, 62]. Havens and Sigward [70] did not examine approach or exit velocities during the COD which is a notable absence because faster approach velocities and minimising velocity loss during cutting has been identified as a key determinant of faster performance [57, 95, 96], and faster approach velocities concurrently elevate knee joint loading [97–101]. Finally, only a limited number of studies have examined the whole-body biomechanical determinants of COD performance using 3D motion analysis [50, 53, 56, 70, 79, 81], but these studies are low in sample size (*n* = 15–34). Therefore, the aim of this study was to expand on previous work [70, 79, 81], by investigating the relationship between cutting biomechanics and cutting performance and surrogates of non-contact ACL injury risk (i.e. KAMs and KIRMs) during 90° cutting with a larger sample size, using a pre-planned cutting task containing a longer approach distance and higher entry velocity. Research has shown COD biomechanics are velocity dependent [33, 97–101], and athletes in multidirectional sport perform high-entry velocity CODs from long approach distances [1, 10, 102]. Conducting such research into the relationship and interaction between performance and injury risk determinants during COD, may assist in the development of more effective ACL injury mitigation and COD speed programmes [78]. It was hypothesised that the mechanical properties responsible for faster performance would concurrently increase knee joint loading.

## Methods

### Research Design

**Fig. 1:**
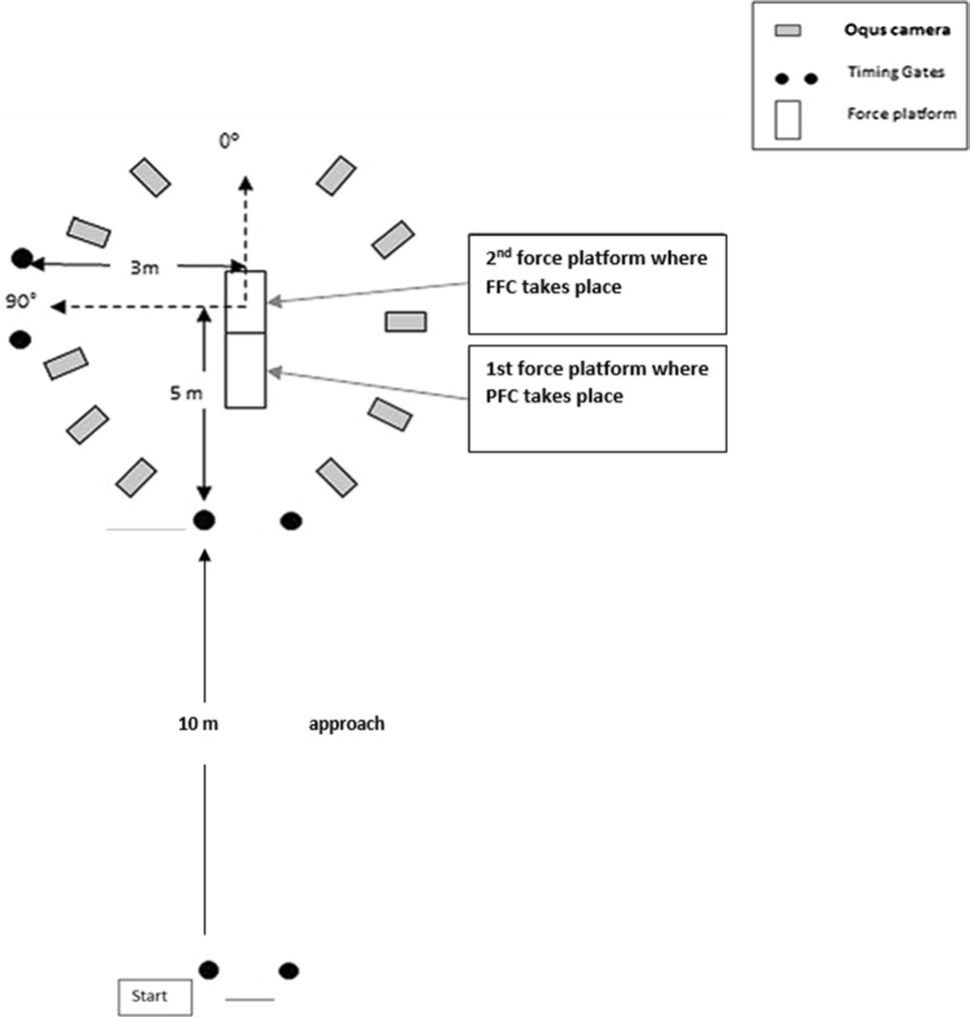
Schematic representations of the 90° cutting task

This study used a cross-sectional design to determine the relationship between COD biomechanics and COD performance (completion time, GCT, exit velocity) and injury risk (peak KAMs and peak KIRMs), following an associative strategy [103]. Participants performed six 90° cuts (Fig. 1) from their right limb and 3D motion and GRF analysis was used to explore the joint kinetic, kinematic, and GRF determinants of performance and injury risk during cutting, similar to the methodological procedures of previous research [70, 104, 105].

### Participants

A minimum sample size of 48 participants was determined from an a priori power analysis using G*Power (Version 3.1, University of Dusseldorf, Germany) [106]. This was based upon a previously reported correlation value of 0.472 (lateral foot plant distance to peak KAM) [70], a power of 0.95, and *α* level of 0.05. Lateral foot plant distance theoretically should be a key variable linked to the performance–injury conflict, because of the requirement to generate medio-lateral impulse [53, 62, 70, 93] for faster performance, and the increased moment arm distance between the GRF vector and knee joint centre increasing peak KAMs, thus ACL injury risk [31–33, 62, 70]. As such, 61 male athletes (mean ± SD; age: 20.7 ± 3.8 years, height: 1.77 ± 0.06 m, mass: 74.7 ± 10.0 kg) from multiple sports (soccer *n* = 43, rugby *n* = 10, cricket *n* = 7, field hockey *n* = 1) participated in this study. For inclusion in the study, all athletes had played their respective sport for a minimum of 5 years and regularly performed one game and two structured skill-based sessions per week. All athletes were free from injury during the study and none of the athletes had suffered a prior traumatic knee injury such as an ACL injury. At the time of testing, players were currently in-season (competition phase). The investigation was approved by the Institutional Ethics Review Board (HSR1617-02), and all participants were informed of the benefits and risks of the investigation prior to signing institutionally approved consent or parental assent documents to participate in the study.

### Procedures

The warm-up [104], marker placement [30, 104, 105, 107], 3D motion analysis [30, 104, 105, 107], and procedures were based on previously published methodologies [81, 104, 105, 107]; thus, a brief overview is provided here. Each participant performed six acceptable trials of a 90° pre-planned side-step cut (Fig. 1) as fast as possible and were provided with standardised footwear to control for shoe–surface interface (Balance W490, New Balance, Boston, MA, USA). Completion time was assessed using two sets of Brower timing lights placed at hip height at the start and finish (Draper, UT, USA). Marker and force data were collected over the PFC and FFC using ten Qualisys Oqus 7 (Gothenburg, Sweden) infrared cameras (240 Hz) operating through Qualisys Track Manager software (Qualisys, version 2.16 (Build 3520), Gothenburg, Sweden) and GRFs were collected from two 600 mm × 900 mm AMTI (Advanced Mechanical Technology, Inc, Watertown, MA, USA) force platforms (Model number: 600900) embedded into the running track sampling at 1200 Hz. Using the pipeline function in visual 3D, joint coordinate (marker) and force data were smoothed using a Butterworth low-pass digital filter with cut-off frequencies of 15 and 25 Hz, based on a priori residual analysis [108], visual inspection of motion data, recommendations by Roewer et al. [109], and to preserve the GRF signal to explore kinetic determinants. Additionally, we have previously reported good agreements (*ρ* = 0.768–0.859) for peak KAM participant ranking between 15 and 25 Hz and matched cut-off frequencies (12–12 Hz, 15–15 Hz, 18–18 Hz) [110]; thus, participants were likely to display similar rankings between conditions. Lower limb joint moments were calculated using an inverse dynamics approach [111] through Visual 3D software (C-motion, version 6.01.12, Germantown, USA) and were defined as external moments and normalised to body mass. Joint kinematics and GRF were also calculated using visual 3D, while GRF braking and propulsive characteristics were normalised relative to body weight, with vertical, anterior–posterior, and medial–lateral corresponding to Fz, Fx, and Fy, respectively.

### Kinetic and Kinematic Variables

A full description of variables along with definitions, abbreviations, and calculations are provided in Supplementary Material 1. Briefly, lower-limb joint moments were calculated over the FFC and lower-limb joint and trunk angles were also calculated and assessed at initial contact, peak, and range of motion of the FFC. Peak and mean GRF braking and propulsive characteristics were also calculated. Weight acceptance (braking) was defined as the point of initial contact to maximum knee flexion and push-off (propulsion) was defined the point of maximum knee flexion to toe-off. PFC braking forces were also assessed for an indication of braking strategies, and horizontal COM velocity profiles at PFC touch-down to determine approach velocity, FFC touch-down, and FFC toe-off to determine exit velocity (Supplementary Material 1) were also examined. COD performance dependent variables were completion time, FFC GCT, and exit velocity, while injury risk dependent variables were peak KAMs and peak KIRMs and were used as surrogates of ACL injury risk [31, 32].

### Statistical Analyses

All statistical analyses were performed in SPSS v 25 (SPSS Inc., Chicago, IL, USA) and Microsoft Excel (version 2016, Microsoft Corp., Redmond, WA, USA). Normality was inspected for all variables using a Shapiro–Wilks test. To explore the biomechanical determinants of performance and injury risk-dependent variables, Pearson’s (for parametric data) and Spearman’s (for non-parametric data) correlations were used, similar to previous research [60, 70]. Correlations were evaluated as follows: trivial (0.00–0.09), small (0.10–0.29), moderate (0.30–0.49), large (0.50–0.69), very large (0.70–0.89), nearly perfect (0.90–0.99), and perfect (1.00) [112]. A correlation cut-off value of  ≥ 0.40 was considered relevant according to Welch et al. [51] who also investigated the biomechanical determinants of cutting performance. Thus, correlations greater than this value are only reported. Stepwise multiple regression analysis was also performed to explore the relationship between the abovementioned variables and key primary performance and injury risk variables. Only significantly correlated variables that were parametric were considered for the Stepwise multiple regression analysis, and no more than 6 variables were inputted into the model to ensure a minimum 10:1 participant to independent variable ratio was present [113]. Statistical significance was defined as *p* ≤ 0.05 for all tests. A minimum of four trials was used for each participant [61], and an average of individual trial peaks for each variable was used [104, 114].

## Results

Descriptive statistics are presented in Table 1 for cutting variables. Pearson’s and Spearman’s correlation values between COD biomechanical variables and cut completion times, GCT, exit velocity, peak KAM, and peak KIRM are presented in Supplementary material 2. FFC GCT and peak KIRM were non-parametric; thus, Stepwise regression analysis could not be performed.

**Table 1:** Cutting biomechanical variables descriptive statistics

Variable	Mean	SD	Variable	Mean	SD
			Braking GRF		
Completion time (s)	1.759	0.135	PFC HBF—pk (BW)	– 1.66	0.40
Sagittal joint moments					
FFC pk HFM (Nm/kg)	– 2.72	0.72	PFC HBF—mean (BW)	– 0.60	0.12
FFC pk KFM (Nm/kg)	3.40	0.68	FFC VBF—pk (BW)	2.56	0.52
FFC pk ADFM (Nm/kg)	– 1.84	0.49	FFC VBF—mean (BW)	1.57	0.18
Sagittal joint moments					
FFC HFA (˚)—pk	47.5	11.2	FFC HBF—pk (BW)	– 1.44	0.33
FFC HFA (˚)—IC	43.1	8.6	FFC HBF—mean (BW)	– 0.85	0.16
FFC HFA (˚)—ROM	4.4	4.6	PFC RBF—pk (BW)	3.06	0.58
FFC KFA (˚)—pk	62.5	7.2	PFC RBF—mean (BW)	1.15	0.18
FFC KFA (˚)—IC	23.1	4.6	FFC RBF—pk (BW)	3.03	0.59
FFC KFA (˚)—ROM	39.4	6.6	FFC RBF—mean (BW)	1.93	0.25
			Propulsive GRF		
FFC ADFA (˚)—pk	78.3	8.2	FFC VPF—pk (BW)	1.82	0.27
FFC ADFA (˚)—IC	55.7	10.5	FFC VPF—mean (BW)	1.20	0.11
FFC ADFA (˚)—ROM	22.5	11.2	FFC HPF—pk (BW)	– 0.81	0.22
Injury risk parameters					
pk KAM (Nm/kg)	1.11	0.39	FFC HPF—mean (BW)	– 0.37	0.11
pk KAA (°)	– 11.6	6.5	FFC MLPF—pk (BW)	1.09	0.20
KAA (°)—IC	2.5	4.9	FFC MLPF—mean (BW)	0.75	0.11
pk KIRM (Nm/kg)	– 0.94	0.44	RPF—PK (BW)	2.24	0.38
pk KRA (°)	– 3.9	9.1	RPF—mean (BW)	1.48	0.17
			Braking ratio		
KRA (°)—IC	– 2.5	10.0	PK HBF ratio	0.90	0.22
Trunk variables					
PFC Sag trunk inclination angle—IC (°)	10.0	4.8	Mean HBF ratio	1.47	0.31
			GCT		
FFC Sag trunk inclination angle—IC (°)	12.7	6.7	PFC GCT (s)	0.202	0.041
Lateral trunk flexion (˚)—IC	– 15.2	7.9	FFC GCT (s)	0.307	0.058
			Velocity profile		
Lateral trunk flexion (˚)—pk	– 26.6	9.9	Approach velocity (m/s)	4.58	0.41
Lateral Trunk flexion (˚)—ROM	11.4	5.8	FFC touch-down velocity (m/s)	3.45	0.37
Hip, pelvis, foot					
Hip rotation angle (°)—IC	15.0	10.5	Exit velocity (m/s)	3.30	0.30
			Change in velocity		
Hip rotation angle (°)—pk	13.4	9.8	Δ PFC velocity (m/s)	– 1.13	0.19
Hip abduction angle (°)—IC	– 24.3	6.3	Δ FFC velocity (m/s)	– 0.90	0.22
Pelvic rotation (°)—IC	33.8	9.3	Approach time (s)	1.980	0.160
IFPA (°)—IC	17.4	10.3			
Lateral foot plant distance (m)—IC	– 0.308	0.051			

*pk* peak, *GCT* ground contact time, *PFC* penultimate foot contact, *FFC* final foot contact, *KAM* knee abduction moment, *KIRM* knee internal rotation moment, *KFM* knee flexion moment, *HFM* hip flexion moment, *ADFM* ankle dorsi-flexion moment, *HFA* hip flexion angle, *KFA* knee flexion angle, *ADFA* ankle dorsi-flexion angle, *RPF* resultant propulsive force, *RBF* resultant braking force, *VPF* vertical propulsive force, *VBF* vertical braking force, *HPF* horizontal propulsive force, *HBF* horizontal braking force, *MLPF* medio-lateral propulsive force, *ROM* range of motion, *IC* initial contact, *BW* body weight, *IFPA* initial foot progression angle, *GRF* ground reaction force

### Completion Times Correlations

**Fig. 2:**
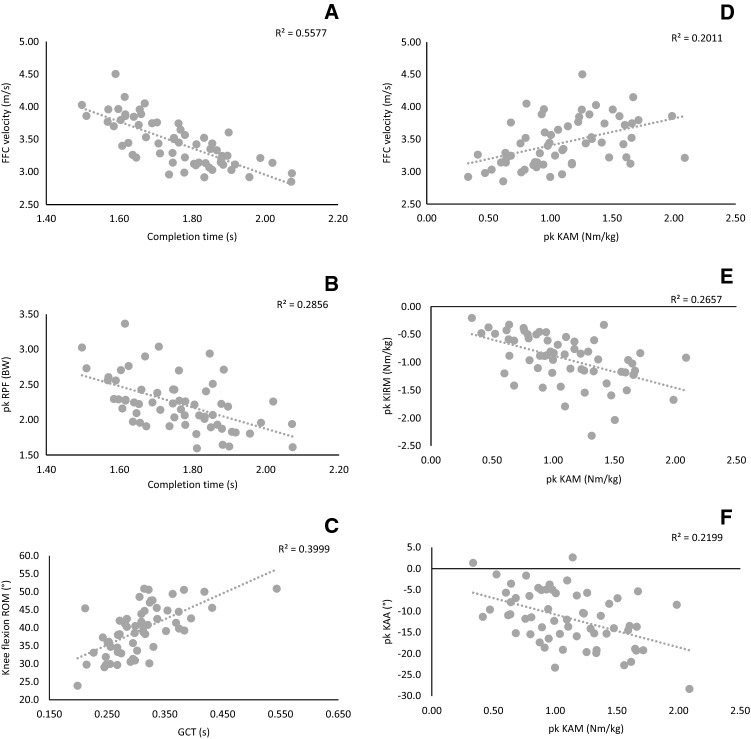
Correlations between change of direction biomechanical variables and performance and injury risk variables. **a** Completion time and FFC touch-down velocity; **b** completion time and peak RPF; **c** GCT and knee flexion ROM; **d** peak KAM and FFC touch-down velocity; **e** peak KAM and Peak KIRM; **f** peak KAM and PEAK KA

Shorter completion times were significantly (*p* ≤ 0.001) and very largely associated with greater FFC touch-down (*ρ* = − 0.752) (Fig. 2) and exit velocities (*r* = − 0.733); largely associated with faster approach velocities (*ρ* = − 0.660), greater peak (*r* = − 0.641) (Fig. 2) and mean resultant propulsive forces (*r* = − 0.530) and medial–lateral propulsive forces (*r* = − 0.588 to − 0.627), shorter approach times (*ρ* = 0.620), greater mean horizontal propulsive forces (*r* = 0.608), greater peak KIRMs (*ρ* = − 0.539), shorter PFC and FFC GCTs (*ρ* = 0.551–0.581), and greater PFC (*r* = 0.551) and FFC mean horizontal braking forces (*r* = 0.535); and moderately associated with greater mean FFC resultant braking forces (*r* = − 0.484), greater peak vertical propulsive forces (*r* = − 0.449) and horizontal propulsive forces (*r* = − 0.460), greater peak KAMs (*r* = − 0.412), greater initial foot progression angles (*r* = − 0.411), and lower hip flexion range of motion (*r* = 0.406). Stepwise multiple regression analysis revealed that greater exit velocities, greater peak resultant propulsive forces, greater PFC mean horizontal braking forces, and greater initial foot progression angles together could explain 64.2% (*r* = 0.801, adjusted 61.6%, *p* = 0.048) of the variation in completion time. The regression equation is presented in Table 2.

**Table 2:** Stepwise multiple regression predictors for completion time, exit velocity and peak KAMs

Block	Variable	*r*	*r*^2^ (%)	Adjusted *r*^2^ (%)	*r*^*2*^ change (%)	Adjusted *r*^2^ change (%)	*B*	Standard error *β*	*β*
Completion time predictors									
1	Exit velocity	0.733	0.538 (53.8)	0.530 (53.0)	0.538 (53.8)	0.530 (53.0)	– 0.237	0.045	-0.536**
2	FFC peak RPF	0.746	0.557 (55.7)	0.541 (54.1)	0.019 (1.9)	0.012 (1.2)	– 0.022	0.036	-0.062*
3	PFC mean HBF	0.785	0.616 (61.6)	0.595 (59.5)	0.059 (5.9)	0.054 (5.4)	0.305	0.106	0.260*
4	IFPA	0.801	0.642 (64.2)	0.616 (61.6)	0.026 (2.6)	0.021 (2.1)	– 0.002	0.001	-0.177*
Exit velocity predictors									
1	FFC peak MLPF	0.651	0.432 (42.3)	0.413 (41.3)	0.432 (42.3)	0.413 (41.3)	0.683	0.179	0.440**
2	FFC KFM	0.690	0.478 (47.8)	0.457 (45.7)	0.052 (5.2)	0.044 (4.4)	0.114	0.050	0.225*
Peak KAM predictors									
1	FFC mean VBF	0.497	0.247 (24.7)	0.234 (23.4)	0.247 (24.7)	0.234 (23.4)	1.017	0.222	0.457**
2	FFC peak KAA	0.652	0.426 (42.6)	0.406 (40.6)	0.179 (17.9)	0.172 (17.2)	– 0.026	0.006	-0.425**

*FFC* final foot contact, *MLPF* medio-lateral propulsive force, *KFM* knee flexion moment, *VBF* vertical braking force, *HBF* horizontal braking force, *PFC* penultimate foot contact, *KAA* knee abduction angle, *IFPA* initial foot progression angle, *RPF* resultant propulsive force

**p < 0.001; *p < 0.05

### FFC GCT Correlations

Shorter GCTs were significantly (*p* ≤ 0.001) and largely associated with greater lateral foot plant distances (*ρ* = 0.626), lower peak knee and hip flexion angles and range of motion (*ρ* = 0.603–0.670) (Fig. 2), and lower peak lateral trunk flexion angles and range of motion (*ρ* = 0.595–0.623).

### Exit Velocity Correlations

Faster exit velocities were significantly (*p* ≤ 0.001) and very largely associated with shorter completion times (*r* = − 0.733) and greater FFC touch-down velocities (*ρ* = 0.725); largely associated with greater mean and peak medial–lateral (*r* = 0.638–0.651) and resultant propulsive forces (*r* = 0.549–0.568), shorter FFC GCTs (*ρ* = − 0.569), greater peak vertical (*r* = 0.540) and horizontal propulsive forces (*r* = 0.500), and greater approach velocities (*ρ* = 0.533); and moderately associated with greater mean vertical propulsive forces (*r* = 0.499), shorter PFC GCTs (*ρ* = − 0.484), greater peak KFMs (*r* = 0.482), lower hip flexion range of motion (*ρ* = 0.470), greater FFC mean vertical braking forces (*r* = 0.456), and greater PFC mean horizontal braking forces (*r* = − 0.430). Stepwise multiple regression analysis revealed that greater FFC peak medial–lateral propulsive forces and peak greater KFMs together could explain 47.8% (*r* = 0.690, adjusted 45.7%, *p* = 0.019) of the variation in exit velocity. The regression equation is presented in Table 2.

### Peak KAMs Correlations

Greater peak KAMs were significantly (*p* ≤ 0.001) and largely associated with greater peak KIRMs (*ρ* = − 0.557) (Fig. 2); and moderately associated with greater FFC touch-down velocities (*ρ* = − 0.491) (Fig. 2), greater peak KAAs (*r* = − 0.468) (Fig. 2), greater FFC mean vertical, horizontal, and resultant braking forces (*r* = 0.434–0.497), and shorter completion times (*r* = − 0.412). Stepwise multiple regression analysis revealed that greater FFC mean vertical braking forces and greater peak KAA together could explain 42.6% (*r* = 0.652, adjusted 40.6%, *p* < 0.001) of the variation peak KAM. The regression equation is presented in Table 2.

### Peak KIRM correlations

Greater peak KIRMs were significantly (*p* ≤ 0.001) and largely associated with greater peak KAMs (*ρ* = − 0.557), greater FFC touch-down velocities (*ρ* = − 0.551), shorter completion times (*ρ* = 0.539), greater approach velocities (*ρ* = − 0.534), and greater FFC peak resultant braking forces (*ρ* = − 0.505); and moderately associated with greater FFC mean (*ρ* = − 0.468) and peak vertical braking forces (*ρ* = − 0.475), and greater FFC peak resultant braking forces (*ρ* = − 0.458).

## Discussion

The aim of this study was to expand on the work of previous research [70, 79, 81] by investigating the relationship between cutting biomechanics and cutting performance and surrogates of non-contact ACL injury risk during a long cutting task, in a large sample size. The results of this study substantiate previous work [70, 79, 81] and the study hypothesis, whereby techniques and mechanics associated with faster performance (i.e. faster cutting COM velocities, greater FFC braking forces in short GCTs, greater KFMs, smaller hip and knee flexion, and greater internal foot progression angles) are in direct conflict with safer COD mechanics (i.e. reduced knee joint loading) (Table 2, Fig. 2), and support the concept that a “performance–injury conflict” exists during cutting [78–80, 101].

From a performance perspective, stepwise multiple regression analysis revealed that greater exit velocity, greater peak resultant propulsive forces, greater PFC mean horizontal braking force, and greater initial foot progression angle together could explain 64.2% (*r* = 0.801, adjusted 61.6%, *p* = 0.048) of the variation in cutting completion time (Table 2). Greater exit velocities permit athletes to cover greater horizontal displacements over shorter times, while greater resultant propulsive forces increase impulse which, based on the impulse–momentum relationship, leads to greater changes in momentum, thus velocity [115, 116]. Therefore, it is unsurprising that the two aforementioned variables were strong determinants of cutting performance. Additionally, cutting is a multistep action [30, 98, 117–119] and displaying greater braking forces in a posteriorly directed direction facilitates reductions in momentum (net deceleration) to permit effective braking [94], thus rationalising the importance of PFC horizontal braking forces for faster cutting performance. Finally, greater internally rotated foot postures reduce the redirection requirements during the COD by more effectively aligning the whole-body COM towards the intended direction of travel [70, 82, 120]. Consequently, these findings highlight that faster 90° cutting performance is underpinned by the interactions between velocity, propulsion, braking, and technique.

To our best knowledge, only three studies have concurrently investigated COD performance and injury risk biomechanical determinants [70, 79, 81]; however, based on these studies and biomechanical principles, mechanics and techniques required for safer cutting performance, thus injury mitigation, are at odds with performance [70, 78–80, 101]. The “performance–injury conflict” is problematic because athletes are unlikely to adopt safer strategies at the expense of faster performance [70]. The results of the current study support the limited research [70, 78–81] and the concept of a “performance–injury conflict” [70, 78–80, 101], whereby techniques and mechanics associated with faster performance (i.e. faster PFC and FFC velocity, greater FFC braking forces over short GCTs, greater KFMs, smaller hip and knee flexion, and greater internal foot progression angles) are in direct conflict with safer COD mechanics (i.e. reduced knee joint loading) (Fig. 2). This issue is problematic for practitioners and athletes who want to adopt cutting strategies that maximise performance while concurrently minimising injury risk. For example, greater COM approach velocities and velocity over key instances of the PFC and FFC were associated with cutting faster performance and greater knee joint loads. Instructing athletes to perform COD actions slowly is not a viable strategy [78, 101], given its importance for faster performance [57, 81, 95, 96]. As such, practitioners must acknowledge that increased knee joint loads are typically associated with greater approach and COD velocity profiles, and should, therefore, progress COD velocity progressively and cautiously with their athletes [101].

Supporting previous work [70, 81], greater peak KFMs were associated with faster cutting performance; likely a product of the faster approach velocities and braking forces. Greater frontal and transverse knee joint loads were moderate to largely associated with greater COM approach velocities, and greater velocity profiles over key instances of the PFC and FFC. These findings support the concept that approach velocity is a key factor regulating cutting knee joint loads [33, 80, 81, 97, 98, 100, 101]. In addition, higher impact braking forces over shorter GCTs were moderate to largely associated with faster performance. Conversely, lower braking forces over longer GCTs were characteristics associated with lower knee joint loads but slower performance. Greater KFMs and posterior GRFs are also associated proximal anterior tibial shear [87, 89] and potential ACL loading [74, 83–85], but are also associated with faster cutting performance [17, 70, 81]; highlighting the conflict between performance and injury risk. Again, braking forces and GCT are influenced by an athlete’s approach velocity and therefore, given its importance for performance, lowering braking forces and increasing GCT duration are not advisable strategies for coaches to implement with their athletes, but they should acknowledge this conflict when coaching cutting.

From a cutting technical perspective, sagittal plane lower-limb kinematics have an important role for performance and injury risk [78, 79]. For example, athletes in the present study who demonstrated faster performance yet greater knee joint loads, demonstrated smaller FFC hip and knee flexion and thus, arguably a “stiffer” hip and knee strategy. This result supports previous work that found increasing knee flexion during cutting concurrently reduced braking GRFs and peak KFMs [80], but negatively impacted performance by increasing GCT and reducing exit velocity. Celebrini et al. [73] found increasing knee flexion reduced KAMs during cutting, while Welch et al. [53] has reported resisting hip flexion over weight acceptance was associated with faster cutting performance. In the transverse plane, greater initial foot progression angles were moderately associated with faster cutting performance and previous work has shown strong relationships between initial foot progression angles and peak KAMs [61, 68], indicating a potential trade-off between performance and injury risk.

A stiffer (i.e. reduced range of motion) hip and knee strategy is effective for performance by reducing GCT and potentially permitting more effective reactive strength and stretch shortening cycle utilisation [53, 121, 122], thus facilitating more effective force transmission due to the rapid transition from braking to push-off. However, stiffer and extended braking strategies ineffectively dissipates forces and energy [80, 123–127], increases loading rates [128], and may increase anterior tibial [74, 83–85] and knee abductor loading [128–131]. Soft weight acceptance strategies are often coached in injury mitigation programmes to reduce impact GRFs and knee joint loads [73, 132–134]; however, practitioners must consider the conflict between performance and injury risk when manipulating such sagittal plane joint kinematics during cutting. Because ACL injuries occur ≤ 50 ms at extended knee postures with minimal hip flexion [26, 135], encouraging greater initially flexed postures with rapid hip and knee co-flexion could be a safer cutting strategy [136], but could be disadvantageous to performance and thus, practitioners should be conscious of this conflict when manipulating sagittal plane mechanics.

Of concern, large relationships were observed between peak KAMs and KIRMs (Fig. 2). This finding is problematic because ACL strain is amplified when a combination of high frontal and transverse knee moments are generated in combination with anterior tibial shear, compared to uniplanar loading [34–38]. The majority of investigations that have investigated the biomechanical determinants of injury risk during COD have primarily focused on KAMs [30, 31, 33, 61–64, 66–71, 79], with only a limited number of studies investigating KIRMs [31, 52, 67, 71, 81]. The results from this study show a large relationship between the peak KAMs and KIRMs; however, greater FFC touch-down velocities and mean vertical braking forces were the only two variables to be moderately related to both peak KAMs and KIRMs. Therefore, it is likely that participants with high frontal knee loads are “worse-off” performers, who also incur high transverse knee loads.

Although several variables have been identified as factors linked to faster performance and greater knee joint loads, some variables have been shown to be associated with faster performance and lower knee joint loads, or offer no associated performance detriments or hazardous increases in knee joint loads (Supplementary Material 2). For example, greater peak KAAs were moderately associated with greater peak KAMs, corroborating previous research [33, 61–64], while no associated performance benefits were found in terms of KAA and cutting performance, which is in line previous research [50]. Increased KAAs have the effect of placing the knee more medial to the resultant GRF vector and, thus, increase the lever arm of the resultant GRF vector relative to the knee joint, leading to an increased KAM [62]. Additionally, increases in knee valgus angle of 2˚ can lead to a 40 Nm change in knee valgus moment [137], while prospective research reported greater valgus angles were associated with increased risk of non-contact ACL injury [77]. As such, reducing KAAs during cutting appears to be viable strategy to reduce knee joint loading, thus ACL injury risk, with no associated cutting performance detriments.

Greater PFC mean horizontal braking forces were largely associated with faster cutting performance, and previous research that has found greater PFC braking forces and PFC dominant braking strategies were associated with faster 180˚ COD performance [57, 58, 88, 94] and lower knee joint loads [57, 58, 88, 94]. During sharper COD, athletes will need to reduce their momentum to perform the COD [57, 95, 138]. Therefore, encouraging greater reductions in COM velocity over the PFC to lower the subsequent velocity at FFC (key determinant of greater knee joint loads), by facilitating effective PFC braking, could also lower knee joint loads while maintaining performance. It is worth noting that some athletes may not effectively adopt a PFC dominant braking strategy, so that they can maintain velocity and transfer it to the exit. This is problematic, however, because this results in higher COM FFC velocities which magnifies knee joint loads (Fig. 2). Thus, encouraging a PFC braking strategy appears to be a practical solution; however, practitioners should be conscious that athletes should be conditioned to be able to generate their own propulsive impulse and momentum during the push-off, so they are not solely reliant on initial momentum. Finally, smaller lateral trunk flexion angles and range of motion were largely associated with smaller GCTs, a critical determinant of faster performance [50, 51, 53–55, 58, 59], while greater lateral trunk flexion angles have been shown to increase knee joint loads [31, 62, 67]. Previous research has shown that medial trunk lean towards the direction of travel was associated with faster performance [50, 53]. Consequently, practitioners should instruct cutting techniques with smaller lateral trunk flexion (trunk lean towards the intended cut) and range of motion for faster and safer performance.

Overall, mechanics associated with faster cutting performance are in direct conflict with mechanics for safer cutting. It is important to note that optimal performance and “high-risk” knee joint loading is not attributed solely to one variable, but the amalgamation and interaction of velocity, joint kinematics and kinetics, and braking and propulsive forces (Table 2). As such, practitioners must consider the performance and injury risk implications when coaching and modifying cutting techniques. In light of the finding that faster athletes generally display greater knee joint loads and are unlikely to sprint slower, it is imperative that athletes have the physical capacity (i.e. neuromuscular control, co-contraction, and rapid force production) and technique to tolerate the knee joint loading demands of side-steps [9, 29, 33, 62, 133, 139–141]. It is likely that physically better conditioned athletes (faster athletes) approach faster potentially due to a “self-regulation” concept, whereby they know they have the physical capacity to tolerate the loads associated with high-velocity COD [57, 96], while better performers may also have the strength capacity to adopt favourable mechanics which contributes to superior cutting performance [56, 96].

Specifically, high levels of strength and activation of the hamstrings [140, 142, 143], gluteals [142, 144], soleus [142, 145], and trunk [72, 146] are needed to reduce non-contact ACL injury risk, support the multiplanar knee joint loads experienced during COD [141, 142, 147, 148], and assist in ligament unloading [141, 147]. This might be best achieved via a periodised multicomponent training programme which integrates strength, plyometric (jump-landing), balance, trunk control, and COD training [133, 149]. Moreover, given the importance of velocity for faster performance, it is integral that practitioners progressively expose athletes to cutting drills of higher velocity [101], and consider the athlete’s training status and strength capacity when exposing them to high-velocity cutting drills [5, 9, 18].

## Limitations

It is worth noting that there were several limitations in the present study. First, males were only investigated, thus caution is advised regarding the generalisation of these results to female athletes and other athletic populations. Second, the biomechanical demands are angle dependent [29, 63, 82, 95, 138, 150–153]; thus, the findings of this study are applicable to pre-planned 90° cuts only. Practitioners should, therefore, be cautious extrapolating these findings to agility tasks because subtle differences in cutting kinetics and kinematics have been observed between pre-planned and unplanned (generic stimuli) cutting [154, 155]. However, it is worth noting that the use of generic stimuli for the unplanned cutting tasks (i.e.., flashing light/arrow) have been criticised because they are not a sport-specific stimulus and lack ecological validity [10, 156, 157]. Further insight is required into biomechanical determinants of performance and surrogates of ACL injury risk in cuts and turns of different angles, actions, and unplanned tasks utilising a sport-specific stimulus. But, notwithstanding this limitation, the findings of this study provide a deeper understanding into the biomechanical demands of pre-planned COD, whereby COD speed provides the mechanical and physical basis for agility [5]. The fundamental biomechanical and movement principles should be similar between planned and unplanned cutting [9]; thus, improvements in the mechanical ability to change direction (i.e. fast mover) may theoretically, and speculatively, transfer to improved agility [5, 158, 159]. While from a motor skill learning perspective, the practice of pre-planned cutting is advocated before increasing intensity, complexity, and sports-specificity with the introduction of unanticipated cutting [5, 9, 17, 18]. Further research is needed exploring the effect of COD speed training on agility performance.

Although a standardised surface was used, this surface does not reflect the grass and artificial field-turfs that the athletes regularly perform their CODs on. Additionally, the present study used a discrete data analysis approach, similar to that of previous work who inspected the relationship between cutting biomechanics and performance and surrogates of ACL injury risk [70, 81]; however, this approach can lead to regional focus bias and potentially valuable information is left unexamined [160, 161]. Therefore, for a deeper level of understanding, future research is necessary that considers the full temporal waveform for further insight into the biomechanical determinants of cutting performance and surrogates of ACL injury risk. Finally, it should be noted that the p-values for correlational analysis were not Bonferroni corrected which could increase type 1 error rate. However, Bonferroni correction is a controversial area which can also increase type 2 error rate, reduce statistical power, and can lead to publication bias, and arguably the magnitude is of greater importance [162, 163]; justifying the use of the ≥ 0.40 threshold approach adopted in the present study as proposed by Welch et al. [51]. Nevertheless, retrospective correlational analysis of the data with Bonferroni correction (p-value multiplied by number of correlations) confirmed that all variables with correlation values ≥ 0.40 still satisfied statistical significance.

## Conclusion

The results of this study confirm that techniques and mechanics associated with faster cutting performance (i.e. faster COM cutting velocities, greater FFC braking forces in short GCTs, greater KFMs, smaller hip and knee flexion, and greater internal foot progression angles) are in conflict with safer cutting mechanics (i.e. reduced knee joint loading) (Supplementary material 2, Fig. 2), and support the “performance–injury conflict” concept [78–81, 101]. Consequently, practitioners must be cautious when coaching and manipulating cutting technique and mechanics, and should acknowledge the implications of technique modification on performance and potential injury risk. Because athletes are driven by performance, techniques and mechanics that result in effective performance even at the expense of greater knee joint loading will inevitably be adopted and will also be a by-product of their sport. Therefore, practitioners should develop their athletes’ physical capacity (i.e. neuromuscular control, co-contraction, and rapid force production) and technique to tolerate and support the knee joint loading demands of side-steps [9, 29, 33, 62, 133, 139–142]. Knee valgus is linked with greater knee joint loads with no associated performance benefits, while PFC braking dominant strategies and minimising lateral trunk flexion are factors associated with faster performance and safer COD mechanics. Therefore, coaching PFC dominant braking strategies and minimising knee valgus and lateral trunk flexion should facilitate effective performance and potentially reduce knee joint loading, thus potential ACL injury risk.

## Supplementary Information

Below is the link to the electronic supplementary material.

Supplementary file1 (DOCX 15 kb)

Supplementary file2 (DOCX 30 kb)

## Declarations

### Data availability

The datasets used and/or analysed during the current study are available from the corresponding author on reasonable request.
