# Supplementary material for: Biomechanical Determinants of Performance and Injury Risk During Cutting: A Performance-Injury Conflict?
Source: Sports Med. 2021 Apr 3;51(9):1983–98. doi: 10.1007/s40279-021-01448-3 (PMC8363537; doi:10.1007/s40279-021-01448-3)
Supplement: Supplementary file 1 — Supplementary file1 (DOCX 15 kb) [file 40279_2021_1448_MOESM1_ESM.docx]

| **Supplementary material 1. Definitions and calculations for kinetic and kinematic variables examined during cutting** | | | | |
| --- | --- | --- | --- | --- |
|  | **Variable** | **Foot contact** | **Abbreviation** | **Definition or calculation** |
| Sagittal plane joint moments | Peak ankle dorsi-flexion, knee flexion, and hip flexion moment | FFC | ADFM, KFM, HFM | Peak external joint moments during weight acceptance using inverse dynamics |
| Sagittal plane joint angles | Ankle dorsi-flexion angle; knee flexion angle; hip flexion angle | FFC | ADFA, KFA, HFA | Derived from the following order of rotations: flexion (+)/extension (-). Angle between two segments |
| Frontal plane injury risk parameters | Peak knee abduction moments | FFC | KAM | Peak external knee abduction moment (+ abduction/- adduction) during weight acceptance of FFC using inverse dynamics. Synonymous with knee valgus moment. |
|  | Knee abduction angle | FFC | KAA | Knee abduction angle (-) during weight acceptance /adduction (+) |
| Transverse plane injury risk parameters | Peak knee rotation moment | FFC | KIRM | Peak external knee rotation moment (+ external/- internal) during weight acceptance using inverse dynamics |
|  | Knee rotation angle | FFC | KRA | Knee rotation angle (- internal/ + external) during weight acceptance |
| GRF | Peak vertical braking force (Fz) | FFC | VBF | Peak normalised VGRF (Fz) value during weight acceptance |
|  | Mean vertical braking force (Fz) | FFC | Mean VBF | Average normalised VGRF (Fz) during weight acceptance |
|  | Peak horizontal braking force (Fx) | PFC and FFC | HBF | Peak normalised HGRF (Fx) value during weight acceptance |
|  | Mean horizontal braking force (Fx) | PFC and FFC | Mean HBF | Average normalised HGRF (Fx) during weight acceptance |
|  | Peak vertical propulsive force (Fz) | FFC | VPF | Peak normalised VGRF (Fz) value during push-off |
|  | Mean vertical propulsive force (Fz) | FFC | Mean VPF | Average normalised VGRF (Fz) during push-off |
|  | Peak horizontal propulsive force (Fx) | FFC | HPF | Peak normalised HGRF (Fx) value during push-off |
|  | Mean horizontal propulsive force (Fx) | FFC | Mean HPF | Average normalised HGRF (Fx) during push-off |
|  | Peak medio-lateral braking force (Fy) | PFC and FFC | MLBF | Peak normalised MLGRF (Fy) value during weight acceptance |
|  | Mean medio-lateral propulsive force (Fy) | FFC | MLPF | Average normalised MLGRF (Fy) during push-off |
|  | Resultant braking force | PFC and FFC | RBF | Calculated using Pythagoras theorem (resultant force = √ ((Vertical force^2^) + Horizontal force^2^)) over weight acceptance |
|  | Resultant propulsive force | FFC | RPF | Calculated using Pythagoras theorem (resultant force = √ ((Vertical force^2^) + (Horizontal force^2^)) over push-off |
|  | Braking force ratio | Between the two contacts | - | FFC braking force / PFC braking force |
|  | Ground contact time | PFC and FFC | GCT | Duration from IC to toe-off |
| Trunk variables | Lateral trunk flexion | FFC | - | Angle of trunk relative to vertical line perpendicular to the pelvis in frontal plane: (0˚) upright / (+) medial trunk flexion away from plant foot/ (-) lateral trunk flexion towards plant foot |
|  | Forward trunk inclination angle | PFC and FFC | - | Angle of trunk relative to a vertical line, (+) forward trunk lean/ (-) backward trunk lean |
| Hip, pelvis, and foot | Hip rotation angle | FFC | HRA | Femur internally rotated (-)/ external rotation (+) |
|  | Hip abduction angle | FFC | - | Hip abduction angle (-) during weight acceptance phase of FFC/ Adduction (+) |
|  | Pelvic rotation | FFC | - | Angle of pelvis in transverse plane relative to global coordinate system. (0˚) straight and perpendicular, (+) rotation towards intended direction of travel (-) rotation away from intended direction of travel |
|  | Lateral foot plant distance | FFC | - | Lateral distance from initial foot contact of foot COM to proximal end of pelvis |
|  | Initial foot progression angle | FFC | IFPA | Angle of foot progression relative to global coordinate system: straight (0°)/inward rotation (+)/outward rotation (-) angle (°) |
| Velocity/COM | Horizontal velocity of COM | PFC and FFC | - | The first derivative of the model COM (combined lower-limb and trunk model) position was computed to derive anterior-posterior (x), vertical (z), and ML (y) over the PFC and FFC. Resultant horizontal plane velocity was calculated using the following formula: √ ((COM vel (x)^2^) + (COM vel (y)^2^)) to provide a “velocity profile” along the path of the participants COM during the cut.  Model COM velocity at PFC touch-down (approach), FFC touch-down, and toe-off in FFC (exit). |
| Key: PFC: Penultimate foot contact; FFC: Final foot contact; COM: Centre of mass; COD: Change of direction; IC: Initial contact; GRF: Ground reaction force; VGRF: Vertical GRF; HGRF: Horizontal GRF; MLGRF: Medio-lateral GRF; vel: velocity | | | | |
