# Supplementary material for: Biomechanical Determinants of Performance and Injury Risk During Cutting: A Performance-Injury Conflict?
Source: Sports Med. 2021 Apr 3;51(9):1983–98. doi: 10.1007/s40279-021-01448-3 (PMC8363537; doi:10.1007/s40279-021-01448-3)
Supplement: Supplementary file 2 — Supplementary file2 (DOCX 30 kb) [file 40279_2021_1448_MOESM2_ESM.docx]

**Supplementary material 2. Correlation matrix between COD biomechanics and performance and injury risk variables**
